# Fungal and Bacterial Endophytes as Microbial Control Agents for Plant-Parasitic Nematodes

**DOI:** 10.3390/ijerph18084269

**Published:** 2021-04-17

**Authors:** K. Kiran Kumar, Surendra K. Dara

**Affiliations:** 1 ICAR-Central Citrus Research Institute, Nagpur 440033, Maharashtra, India; Kiran.Kommu@icar.gov.in; 2 University of California Cooperative Extension, 2156 Sierra Way, Ste. C, San Luis Obispo, CA 93401, USA

**Keywords:** endophyte, bacteria, fungi, plant-parasitic nematode, bionematicide

## Abstract

Endophytes are symbiotic microorganisms that colonize plant tissues and benefit plants in multiple ways including induced systemic resistance to biotic and abiotic stresses. Endophytes can be sustainable alternatives to chemical nematicides and enhance plant health in a variety of cropping and natural environments. Several in vitro and in vivo studies demonstrated the potential of multiple species of *Fusarium* and *Bacillus* against plant-parasitic nematodes in horticultural, agricultural, and fodder crops and in forestry. While there were efforts to commercialize some of the endophytes as bionematicides, a lack of good formulations with consistent field efficacy has been a major hurdle in commercializing endophytes for nematode control. Identification of efficacious and environmentally resilient strains, a thorough understanding of their modes of action, interactions with various biotic and abiotic factors, and developing strategies that improve their effectiveness are critical areas to advance the commercialization of bionematicides based on fungal and bacterial endophytes.

## 1. Introduction

Plant-parasitic nematodes (PPN) such as root-knot nematodes (*Meloidogyne* spp.), cyst nematodes (*Heterodera* spp. and *Globodera* spp.), and root lesion nematodes (*Pratylenchus* spp.) are important pests of many crops around the world causing significant yield losses. They primarily attack the root system by forming feeding sites such as, single giant cells, syncytia, non-hypertrophied nurse cells and coenocytes, which provide a protective feeding environment [1]. PPN feeding causes root deformity, stunting of plants, yellowing of leaves, and yield reduction. Additionally, PPN also act as entry ways for secondary pests and pathogens by causing wounds in the plant roots [2]. It is estimated that PPN cause an average yield loss of 12.6% equal to USD 215.8 billion in 20 major commercial crops including banana, coconut, peanut, potato, rice and sugarcane around the world [3]. While the root-knot and cyst nematodes are sedentary, root lesion nematodes, the burrowing nematode (*Radopholus similis*), the stem nematode (*Ditylenchus dipsaci*), and the reniform nematode (*Rotylenchulus reniformis*) are migratory [4]. Species of *Longidorus*, *Xiphinema*, *Trichodorus*, and *Paratrichodorus* are also vectors of plant viruses in addition to their feeding damage [5]. Fumigants and various classes of chemical nematicides are commonly used by growers for suppressing PPN, which have potential negative impacts on the environment including the detrimental effect on beneficial soil microbiota. Due to the higher cost of fumigation and the need for effective and safe non-chemical alternatives, bionematicides are gaining interest in the recent years [6,7].

There have been reports of nematode suppressive soils where PPN populations are suppressed due to the presence of bacteria and fungi. These beneficial microorganisms limit PPN proliferation by certain trapping structures or by producing toxins. *Trichoderma harzianum, Purpureocillium lilacinum, Pochonia chlamydosporia, Monacrosporium lysipagum, Pseudomonas fluorescens* and *Pasteuria penetrans* are some of the microorganisms involved in nematode suppression [8,9,10,11]. Bionematicides based on the fungi *P. chlamydosporia*, *P. lilacinum*, *Arthrobotrys oligospora*, *Trichoderma* spp., and *Verticillium* spp., and bacteria *P. fluorescens*, *P. penetrans*, and *Bacillus* spp. have been used in many cropping systems including lettuce-tomato, tomato-carrot, potato, common bean, tomato, cucumber, snapdragon, wheat and sugarcane [12,13,14,15,16,17,18,19]. While these bacteria and fungi appear to be effective in PPN suppression, there is an increased interest to explore endophytes as bionematicides especially for sedentary PPN because both of them colonize the same plant tissues and the former have a better chance of suppressing the latter [20]. Since many endophytes produce secondary metabolites that have pesticidal properties, they could be excellent candidates as bionematicides [21]. This review provides an overview of bacterial and fungal endophytes as potential bionematicides, and the challenges and opportunities in associated with their commercialization.

## 2. Endophytes

Endophytes are microorganisms that colonize plant tissues and live between plant cells in a symbiotic relationship usually characterized by mutualism. Endophytes include members of bacteria, fungi, archaea, and protists, but bacteria and fungi are the most common and widely studied taxa. While the plants provide living space and nutrition to the endophytes, the latter trigger immune responses in plants that help them withstand various biotic and abiotic stresses. Since some of the endophytes are closely related to plant pathogens, but are avirulent, plants sense their presence and activate defense responses through the production of various proteins, secondary metabolites, and hormones imparting tolerance to pathogens, herbivores, and abiotic stresses such as salinity and drought. Endophytes are in rhizosphere and phylloplane and colonize plants at various stages through seeds, seedlings, or vegetative propagative materials. The species composition, population abundance, and colonization depend on the plant species and soil and environmental conditions. While the endophytism is a common phenomenon studied for a long time, the impact of endophytes on pests including PPN is a new area of scientific interest particularly due to the demand for environmentally sustainable agricultural practices. Sikora et al. [20] discussed various laboratory, greenhouse, and field studies that explored endophytes for controlling PPN.

### 2.1. Fungal Endophytes

Among other endophytes, fungal endophytes are the most common, diverse, and well-studied group for their role in imparting stress resilience to plants [22,23]. Fungal endophytes are categorized into clavicipitaceous and non-clavicipitaceous groups based on their taxonomy, evolutionary relatedness, ecology, and host range [24,25,26,27]. Calvicipitaceous endophytes include the genera *Balansia*, *Balansiopsis*, *Atkinsonella*, *Echinodothis*, *Epichloe*, *Myriogenospora*, *Neotyphodium*, and *Parepichloe*, which are commonly associated with grasses and rely on their host throughout their life cycle as mutualists [24,26,28,29]. These endophytes grow in the intercellular spaces of the aboveground plant tissues and are transmitted both horizontally and vertically depending on the species [27,30]. Non-clavicipitaceous endophytes such as *Fusarium*, *Colletotrichum*, *Phomopsis*, and *Xylaria* are found in most terrestrial plants and do not rely on plants to complete their life cycle [29,31].

Among various species, *Fusarium oxysporum* is the most dominant endophyte isolated from different plants and is an antagonist of fungal pathogens, insects, and PPN [32,33,34]. Antagonism of *F. oxysporum* towards *Helicotylenchus multicinctus*, *Meloidogyne incognita*, *Meloidogyne graminicola*, *Pratylenchus goodeyi*, and *R. similis*, in banana, melons or tomato has been reported from different studies (Table 1)[35,36,37,38,39,40,41]. Other examples include *Epichloe coenophiala* (=*Acremonium coenophialum*, *Neotyphodium coenophialum*) against *Pratylenchus scribneri*, *Meloidogyne marylandi*, and *Helicotylenchus pseudorobustus* in tall fescue [42,43,44] and *Chaetomium globosum* towards *M. incognita* in cotton [45].

### 2.2. Bacterial Endophytes

Bacterial endophytes are the second most common endophytes and colonize most plant species locally or systemically living within the cells, in the intercellular spaces or vascular system [71,80]. Their antagonism towards PPN has been reported since mid-1990s [71,81]. Most of the Gram-negative endophytes and some species of Gram-positive endophytes are antagonists of plant pathogens [82]. *Agrobacterium radiobacter*, *Burkholderia cepacian*, and *P. fluorescens* are examples of Gram-negative endophytes and *Bacillus* spp. are examples of Gram-positive endophytes. It has also been found that species of *Acrhomobacter*, *Acinetobacter*, *Agrobacterium*, *Bacillus*, *Brevibacterium*, *Microbacterium*, *Pseudomonas*, *Xanthomonas*, and others have the potential for controlling PPN [83].

### 2.3. Mode of Action of Endophytes

Endophytes suppress the growth and development of various stages of PPN through various mechanisms and thus contribute to the improvement of plant health. These can be categorized into direct and indirect mechanisms as described below:

#### 2.3.1. Direct Mechanisms

Endophytes can directly attack, kill, immobilize or repel PPN as they find their host, compete for space and produce secondary metabolites and other compounds that are detrimental to PPN. Endophytes produce metabolites such as flavonoids, peptides, quinones, alkaloids, steroids, phenols, terpenoids, and polyketones or lytic enzymes such as chitinases, cellulases, hemicellulases, and 1,3-glucanases [84,85,86]. These compounds inhibit the growth and development of PPN and other biotic stressors through antibiosis. Additionally, endophytes occupy plant tissues and inhibit PPN through niche competition or competitive displacement [85]. For example, *F. oxysporum* isolated from banana, paralyzes and kills *P. goodeyi* [39], while *C. globosum* produce secondary metabolites viz., chaetoglobosin A, chaetoglobosin B, flavipin, 3-methoxyepicoccone and 4,5,6-trihydroxy-7-methylphthalide against *M. incognita* [87].

#### 2.3.2. Indirect Mechanisms

Endophytes induce systemic resistance by upregulating genes that produce various phytohormones, phytoalexins, volatile organic compounds, pathogenesis-related proteins, and trigger salicylic acid, jasmonic acid, and ethylene pathways that protect plants from stressors. Some of these defenses antagonize stressors such as PPN while others such as phytohormones promote plant growth and compensate for the damage by stressors. Additionally, endophytes can also influence root exudate composition and production that further inhibit stressors. For example, *Bacillus sphaericus* B43 and *Rhizobium etli* G12 application induced systemic resistance to *Globodera pallida* in potato [88] and to *M. incognita* in tomato [89]. *Meloidogyne incognita* showed a preference to root exudates extracted from tomato roots than those extracted from roots colonized by endophytic *F. oxysporum* strain Fo162 [35]. Such an interaction was also observed in *M. graminicola* with respect to rice in the presence or absence of *F. moniliforme* strain Fe14 [74].

## 3. Endophytes as Bionematicides

While regulatory guidelines might place certain endophytes as biostimulants or soil amendments and others as biopesticides, for this review, we present various examples of endophytes as microbial control agents of PPN in a variety of crops and forestry.

### 3.1. Vegetable Crops

Researchers around the world evaluated the culture filtrates of bacterial and fungal endophytes against root-knot nematodes in cucumber, okra, and tomato with a majority of the studies in tomato (Table 1) [35,46,47,50,51,52,53,54,55]. Tian et al. [53] reported that *Acremonium implicatum* inhibited the formation of root galls caused by *M. incognita* in potted tomato plants to 41 per plant compared to 122 in untreated plants. Under field conditions, root gall index was significantly reduced to 25 from the endophyte treatment compared to 96 in untreated plants. In a greenhouse study, Hu et al. [54] found that various isolates of *Bacillus cereus* caused 53–76% reduction in *M. incognita* egg masses, 70–81% reduction in gall formation while repelling second stage juveniles (J2s). Similarly, isolates of *Bacillus* sp., *Methylobacterium* sp., and *Pseudomonas* sp. significantly reduced the egg masses, number of eggs per egg mass, number of adult females, and overall *M. incognita* numbers in both soil and roots of tomato growing in pots [55]. The two isolates of *Bacillus* sp. resulted in the lowest gall index of 1.33 compared to 4.67 in control plants. Zhao et al. [90] reported 88–93% mortality in *M. incognita* J2s and 88–83% reduction in egg-hatch from *Pseudomonas protegens* and *Serratia plymuthica* in greenhouse tomato. Metabolites of both bacteria reduced the number of egg masses and root galls, while *P. protegens* also promoted seed germination and plant growth. In another study, Munif et al. [78] obtained 81% mortality of J2s and 81% reduction in egg-hatch of *M. incognita* with a consortium of bacterial endophytes isolated from trees. Details of these endophytic species were not revealed, however.

Seed treatment with endophytes has been explored as an option for controlling PPN. Yan et al. [49] found that *Acremonium* sp., *Chaetomium* sp., *Fusarium* spp., *Paecilomyces* sp., *Phyllosticta* sp., and *Trichoderma* sp. reduced the number of galls by *M. incognita* in cucumber after treating the seeds. Metabolites from some of these endophytes negatively impacted the motility of J2s. Among these fungi, *Chaetomium* sp. had the highest colonization of roots (70.5%) and aboveground parts (73.5%) with 42–47% reduction in root galls. In another study, bacterial isolates of *Pantoe aagglomerans*, *Cedecea davisae*, *Enterobacter* spp., and *Pseudomonas putida* reduced early root penetration of *M. incognita* in tomato by up to 56% when applied as seed treatment, root dip, or soil drench [51]. Seed treatment followed by soil drenching appeared to be more effective than single application in reducing gall formation.

Some consider that a consortium of beneficial microorganisms is better than using a single microorganism and several commercial products based on microbial consortia have been developed in the recent years [78,91]. However, consortia may not always provide better control than a single endophyte due to potential negative interactions among the members. For example, the fungal endopyte *Piriformospora indica* alone suppressed *M. incognita* infestations in tomato better than as a member of a consortium with *Bacillus pumilus* and *P. fluorescens* [92]. While all of them promoted the plant growth, nematode control of *P. indica* decreased in the presence of bacterial endophytes.

### 3.2. Fruit Crops

In pineapple, application of *Bacillus* sp. isolated from its roots suppressed *R. reniformis* infestations by 54–60% in two cultivars [93]. Crop rotation with sunn hemp (*Crotalaria juncea*) and application of the endophyte were found to be effective non-chemical alternatives for PPN control in pineapple. Effective use of *F. oxysporum* for controlling PPN in banana has been reported from several studies. *Fusarium* isolates induced systemic resistance and caused up to 41% of reduction in *R. similis* J2s penetration 15 days after treatment in a greenhouse study [59]. Three strains of *F. oxysporum* caused >85% mortality of *R. similis*, *P. goodeyi* and *H. multicinctus* mixed stages (J2s, males and females) after 24 h of exposure [41]. A strain of *Streptomyces* sp. isolated from banana roots showed >50% inhibiting rate of *Meloidogyne javanica* J2s in vitro and 71% of biocontrol efficiency in sterile soil [63]. However, in the presence of *Streprtomyces* sp., populations of bacterivorous nematode genera *Mesorhabditis* and *Cephalobus* also increased. In another study, Mendoza and Sikora [61] found that the combination of the strains of *F. oxysporum* and the egg parasitic fungus *P. lilacinum* (=*Paecilomyces lilacinus*) reduced *R. similis* populations by 68.5% while the combination of *F. oxysporum* and *Bacillus firmus* reduced PPN populations by 86% in banana under greenhouse conditions.

### 3.3. Tuber Crops

In potato, *A. radiobacter* and *B. sphaericus* induced systemic resistance against the potato cyst nematode, *G. pallida* [94]. Another study demonstrated significant control of *M. incognita* in potato from *R. etli* G12 application with 34% fewer galls than in control [64]. *Bacillus carotarum*, *B. cereus*, and *Pseudomonas pseudoalcaligenes* isolated from the roots and tubers of potato caused ≥80% mortality of *Globodera rostochiensis* J2s in vitro but did not limit the egg-hatch [66].

### 3.4. Ornamental Crops

There was only one example of using endophytes for PPN control in ornamental crops. Application of the endophytic bacteria *P. agglomerans* (MN34) and *P. putida* (MN12) reduced the gall index from *M. incognita* and promoted plant growth in natal plum (*Carissa macrocarpa*), dwarf lilyturf (*Ophopogan japonicas*), and other ornamental plants [67].

### 3.5. Plantation Crops

Endophytes for PPN control have been explored in black pepper, coffee, and other crops. In an Indian study, *B. megaterium* and *Curtobacterium leteum*, isolated from black pepper, provided the highest suppression of *R. similis* J2s among other isolates tested [68]. Similarly, a Vietnamese isolate of *B. megaterium* reduced numbers of *Meloidogyne* sp. by 82 and 73% in soil and pepper roots, respectively [69]. In Indonesia, 52% suppression in root galls and 75% suppression in *M. incognita* numbers was observed from the application of endophytic bacteria isolated from pepper plants [7]. When organic matter, in the form of cow manure, was applied along with endophytic bacteria, there was a 93% decrease in root galls and 97% decrease in nematode populations while a commercial organic fertilizer applied with endophytes caused 86 and 79% reduction in root galls and nematode numbers, respectively. In Vietnam, *Streptomyces* sp. (CBG9) isolated from robusta coffee in Central Highland inhibited *M. incognita* egg-hatch by 86% and caused 85% of mortality in J2s [70].

### 3.6. Agricultural Crops

*Meloidogyne graminicola* is an important pest of rice in South Asia and South East Asia. Rice is a staple food of billions around the world and *M. graminicola* is one of the pests that cause significant losses, if left uncontrolled. Various studies demonstrated the potential of endophytes in controlling *M. graminicola*. In Taiwan, *B. megaterium*, isolated from rice growing regions, provided more than 40% reduction in J2 penetration and gall formation by *M. graminicola* and 60% reduction of J2 migration [72]. In a German study, *Fusarium* isolates from rice producing areas in Vietnam reduced gall formation by 29–42% and improved the root weight by 33% [73]. *Trichoderma* spp. evaluated in this study also had a similar impact with up to 38% reduction in root galling. Similarly, in another German study *F. moniliforme* (Fe14) suppressed J2s penetration by 55% and increased the male to female ratio by 9 times [74]. Additionally, root exudates from endophyte treated plants were also less attractive to J2s.

In the US, *C. globosum* (TAMU 520), isolated from upland cotton used as a seed treatment, negatively impacted *M. incognita*, the cotton aphid (*Aphis gossypii*), and the beet armyworm (*Spodoptera exigua*) [45]. Endophyte reduced nematode infection and female reproduction as well as the fecundity of both insect species. In a different study in the USA, hundreds of nematophagous fungal endophytes were isolated from the roots of corn and soybean where the majority was *Fusarium* spp. along with less common *Hirsutella rhossiliensis*, *Metacordyceps chlamydosporia*, and *Arthrobotrys iridis* [95]. It was also observed that PPN population density influences the endophyte diversity as the fungal diversity in soybean roots was higher when *Heterodera glycines* density was higher.

### 3.7. Fodder Crops

Species of *Epichloë* (=*Neotyphoidium* and *Acremonium*) are endophytes of Gramineae family that contains tall fescue, perennial rye grass and others used for fodder. Although some endophytes release metabolites that are toxic to cattle, there are several examples of PPN reduction from the presence of various species of endophytes (Table 1). An earlier report indicated that the presence of the fungal endophyte *E. coenophiala* significantly reduced populations of *P. scribneri* and *Tylenchorhynchus acutus* in tall fescue and improved the yields [42]. However, endophyte colonized grass appeared to be toxic to cattle because of the ergot alkaloids they release. Inoculating tall fescue with non-ergotic strain of *E. coenophiala* offered resistance to *Pratylenchus zeae* and *P. scribneri*, however, to a lesser extent than ergotic strains [75]. In another study, 100% mortality of *M. marylandi* was observed when PPN-infested soil in pots was inoculated with *E. coenophiala* and with water deficit stress [44].

### 3.8. Forestry

The pine wilt nematode, *Bursaphelenchus xylophilus*, is a serious problem in Korea, Canada, USA, China, Japan and other South Eastern countries causing pine wilt of coniferous trees of the genus *Pinus*. Ponpandian et al. [79] isolated and investigated hundreds of bacterial endophytes for controlling *B. xylophilus*. Among them, *Stenotrophomonas* spp. and *Bacillus* spp. were very effective, causing ≥70% mortality of adult nematodes. Liu et al. [77] isolated 48 genera of Gamma-proteobacteria and others from four species of pine trees in Korea. Among those isolates, *Escherichia coli* and *Serratia marcescens* produced nematicidal metabolites causing 67 and 60% mortality in *B. xylophilus*, respectively, in laboratory assays.

## 4. Metabolites from Endophytes

Endophytes produce an array of secondary metabolites, which have biopesticide potential against plant pathogens and PPN. These metabolites categories include alkaloids, flavonoids, peptides, phenols, polyketones, quinols, terpenoids, and steroids [96], which induce systemic resistance in plants against various biotic and abiotic stresses.Several of these secondary metabolites have nematicidal properties and can be important IPM options (Table 2). For example, *F. oxysporum* isolated from the roots of the tomato cultivar Moneymaker produces several bioactive compounds including 4-hydroxybenzoic acid, indole-3-acetic acid, and gibepyrone D, which are nematicidal with LC_50_ values of 104, 117, and 134 µg/mL, respectively [97]. In another study, five metabolites—chaetoglobosin A, chaetoglobosin B, flavipin, 3-methoxyepicoccone, and 4, 5, 6-trihydroxy-7-methylphthalide—derived from *C. globosum* caused moderate to high levels of mortality in *M. javanica* J2s [87]. With LC_50_ values of 200 µg/mL, chaetoglobosin A and B significantly suppressed nematode reproduction and promoted plant growth in a potted plant study.

## 5. Commercialization

Identification, mass-production, and commercialization of nematicidal endophytes have a great potential for PPN control in many systems in a sustainable manner. Since some of these endophytes induce systemic resistance against multiple stressors or antagonize multiple categories of pests, endophytes can be comprehensive tools for improving overall crop health, yields, and optimize the cost of crop care. Although numerous studies demonstrated the potential of bacterial and fungal endophytes for controlling PPN, there are noncommercial nematicides based on these endophytes. However, some strains are available as biostimulants or biofungicides.

## 6. Constraints and Future Opportunities

Major constraints of biopesticides are their high cost of production and relatively fewer efficacy data compared to synthetic pesticides. As a result, there is some hesitation in the farming community to use biopesticides as their primary choice. Some of the reasons for low, slow, or inconsistent field performance of some endophytes are adverse soil conditions, competition with other soil microorganisms, the presence of mycophagous or bacteriophagous nematodes [105,106]. In some cases, endophytes might release toxic metabolites that are harmful to herbivorous mammals or alter the microbial community in the rhizosphere or phylloplane with negative consequences [107].

Some strategies to improve the prospects of bionematicides based on endophytic bacteria and fungi are investing in applied research that generates field efficacy data, developing formulation technologies that optimize production costs and improve product efficacy, and educating the farming community with IPM practices that make the best use of bionematicides and other such products. Research, outreach, communication at various levels, employing various control options, and exploiting modern technologies for monitoring crop health and delivering agricultural inputs are various elements of IPM [108]. When bionematicides are integrated with other management practices, they provide effective and long-term suppression of PPN.

## Figures and Tables

**Table 1:** Effect of fungal and bacterial endophytes against plant-parasitic nematodes (PPN) in different crops.

PPN Species	Crop	Endophytic Organism	Effect on PPN	References
Vegetable crops				
*Meloidogyne incognita*	Tomato	*Pantoe agglomerans* (MK-29), *Cedecea davisae* (MK-30), *Enterobacter intermedius* (MK-42), *Pseudomonas putida* (MT-19), *P. putida* (MT-04), *Pseudomonas fluorescens* (MK-35)	Reduced the number of galls by 27–43% after soil drench application and reduced nematode infestation as a seed treatment	[46]
*M. incognita*	Tomato	*F. oxysporum* (strain 162)	Reduced nematode penetration by 36–56%	[35]
* M. incognita *	Tomato	* Agrobacterium radiobacter, Bacillus pumilus, B. brevis, B. megaterium, B. mycoides, B. licheniformis, Chryseobacterium balustinum, Cedecea davisae, Cytophaga johnsonae, Lactobacillus paracasei, Micrococcus luteus, Micrcoccus halobius, Pseudomonas syringae and Stenotrophomonas maltophilia. *	Reduced the number of galls and egg masses by 33 and 39%	[47]
*M. incognita*	Bhendi	*Pseudomonas* spp. (EB3) *Bacillus* spp. (EB16, EB18), *Methlobacterium* spp. (EB19)	Reduced the number of adult females, egg masses, eggs per egg mass and lowered root gall index	[48]
*M. incognita*	Cucumber	*Phyllosticta* (Ph5110), *Chaetomium* (Ch1001), *Acremonium* (Ac985), *Paecilomyces* (Pa972)	Reduced the number of galls by 24–58% in the first screening and 15.6–44.3% in the repeated test. *Chaetomium* showed the highest potential for seed treatment against *M. incognita*	[49]
*M. incognita*	Tomato	*Fusarium oxysporum* (Fo162); *Rhizobium etli* (G12)	Reduced the number of eggs per female 35 days after nematode inoculation	[50]
*M. incognita*	Tomato	*P. agglomerans* (MK-29), *C. davisae* (MK-30), *Enterobacter* spp. (MK-42), *P. putida* (MT-19)	Reduced early root penetration of J2s into roots up to 56% when applied as a root dip and soil drench; Reduced the number of galls by seed treatment with endophytic bacteria followed by soil drench application	[51]
*Meloidogyne* spp.	Tomato	*Gliocladium* spp.	Significant decrease in damage intensity to 33% by inoculating conidial suspension at the rate of 10^6^ mL^−1^	[52]
*M. incognita*	Tomato	*Acremonium implicatum*	96.0% of J2s were killed by a culture filtrate after 48 h; Formation of root galls was inhibited in potted plants and root gall index was reduced in the field	[53]
*M. incognita*	Tomato	*F. oxysporum*; *F. solani;* *Trichoderma asperellum*	Reduced nematode penetration; *T. asperellum* and *F. oxysporum* isolates reduced nematode egg densities by 35–46%	[36]
* M. incognita *	Tomato	*Bacillus cereus* (BCM2)	Reduced gall and egg mass indexes	[54]
*M. incognita*	Tomato	*Bacillus* sp. (EB16, EB18) *Methylobacterium* sp. (EB19) *Pseudomonas* sp. (EB3)	Reduced the number of adult females, egg masses, eggs per eggmass, soil and root population of *M. incognita*	[55]
Fruit crops				
*Radopholus similis*	Banana	*Fusarium*	Reduced the number of J2s per gram root by >80%	[56]
*R. similis*	Banana	*F. oxysporum*	Reduced nematode population density on tissue culture plantlets by 49–79%	[37]
*R. similis*	Banana	*Fusarium* spp. (V5w2)	Decreased nematode reproduction by 22.9 and 60.6% in cultivars, Enyeru and Kibuzi respectively	[57]
*M. incognita, Pratylenchus coffeae*, *R. similis, Helicotylenchus multicinctus*	Banana	*Bacillus subtilis* (EPB 5, 22, 31 and EPC 16) Talc based	Reduced nematode population in the combined treatment of EPB 5+31	[58]
*R. similis*	Banana	*F. oxysporum* (S9, P12)	63% reduction in *R. similis* population in root system	[38]
*R. similis*	Banana	*F. oxysporum*	Pre-inoculation of banana plantlets on one half of the root system significantly reduced root penetration of J2s on the non-treated half of the root by 30–40%	[59]
*R. similis*	Banana	*F. oxysporum* (V5w2)	Disrupted nematode reproduction	[60]
*R. similis*	Banana	*F. oxysporum* (strain 162), *Paecilomyces lilacinus* (strain 251), *Bacillus firmus*	Reduced nematode density by 68% after combined application of *F. oxysporum* and *P. lilacinus*; Application of *F. oxysporum* and *B. firmus* resulted in reduced J2 density by 86.2%	[61]
*Pratylenchus goodeyi*	Banana	*F. oxysporum*	Increased paralysis and mortality of motile stages by 17–26% and 62–73% respectively	[39]
*M. incognita*	Squash and melon	* F. oxysporum * (strain 162)	Reduced early root penetration of J2s in squash and melon up to 69 and 73%, respectively	[40]
*R. similis*, *P. goodeyi*, *H. multicinctus*	Banana	*F. oxysporum*	Higher nematode mortality after 24 h exposure to culture filtrates; *H. multicinctus* was less sensitive to culture filtrates than *R. similis* and *P. goodeyi*	[41]
*P. goodeyi*	Banana	*F. oxysporum* (4MOC321, 11SR23)	Significant reduction of *P. goodeyi* population by >50% and percentage root necrosis was reduced by >30%	[62]
* M. javanica *	Banana	* Streptomyces * sp.	Inhibition rate of >50% * in vitro * and biocontrol efficiency of 70.7% in sterile soil against J2s	[63]
Tuber crops				
*M. incognita*	Potato	*R. etli* (G12)	The no. of galls on roots was 34% lower than control	[64]
*Globodera rostochiensis*	Potato	*P. fluorescens*, *P. putida 3*, *P. syxantha*, *P. aurantiacea* 13	Reduced nematode multiplication by 40.7–42.2% over the control with *P. putida* 3 and *P. aurantiacea* 13 respectively	[65]
*G. rostochiensis*	Potato	*Bacillus carotarum*, *B. cereus*, and *Pseudomonas pseudoalcaligenes*	Increased the mortality of J2s by 67–97%; No effect on eggs; suppressed the number of cysts by 51–65% and J2s by 48–76% in greenhouse experiment	[66]
Ornamental crops				
*M. incognita*	Ornamentals	*P. agglomerans* (MN34); *P. putida* 9MN12)	Decreased galling index	[67]
Plantation crops				
*R. similis*	Black pepper	*Bacillus megaterium* (BP 17) and *Curtobacterium luteum* (TC 10)	Higher nematode suppression with *C. luteum* followed by *B. megaterium*	[68]
*Meloidogyne* sp.	Black pepper	*B. megaterium* (DS9)	Reduced nematode population with great inhibition values of 81 and 73%	[69]
*Meloidogyne* spp.*; Pratylenchus* spp.*; Apratylenchus* spp.*; Criconemella* spp.; *Xiphinema* spp.*; Rotylenchulus* spp.	Coffee	*Bacillus* spp., *Serratia* spp., *Paenibacillus* spp., *Enterobacter* spp. and *Streptomyces* spp. (CBG9)	*Streptomyces* sp. showed inhibited egg hatching by 85% and mortality of *M. incognita* J2s by 85%	[70]
*M. incognita;* *R. similis*	Black pepper	AA2, MER7, ANIC, TT2, MER9, HEN1, EH11, TT2	Reduced the number of root galls by 30–91%; reduced nematode population in the soil by 15–99%	[7]
Agricultural crops				
*M. incognita*	Cotton		Reduced 30–50% of root galls by seed treatment application	[71]
*Meloidogyne graminicola*	Rice	*Bacillus megaterium*	Reduced nematode penetration and gall formation by >40%	[72]
*M. graminicola*	Rice	*Fusarium* spp.	Reduced root-galling by 29–42% and increased root weight by 33%	[73]
*M. incognita*	Cotton	*Chaetomium globosum* TAMU 520	Inhibited nematode infection and reduced female production	[45]
*M. graminicola*	Rice	*Fusarium moniliforme* Fe14	Reduced J2 penetration into roots by 55% and increased male to female ratio by nine times.	[74]
Fodder crops				
*Pratylenchus scribneri*	Tall fescue	*Epichloe coenophiala*	Reduced nematode population	[42]
*Meloidogyne marylandi*	Tall fescue	*E. coenophiala*	Reduced the emergence of J2s, number of egg masses per pot and the number of eggs per egg mass	[43,44]
*P. scribneri;* *Helicotylenchus pseudorobustus; M. marylandi*	Tall fescue	*E. coenophiala*	Hinderance in reproduction of the nematodes	[43]
*Pratylenchus* spp.	Tall fescue	*E. coenophiala*	Non-ergot strain AR584 confer resistance in cv. Georgia 5	[75]
*Tylenchorhynchus* spp., *Criconemella* spp., *Helicotylenchus* spp.*; Pratylenchus* spp.	Tall fescue	*E. coenophiala* (AR584; AR542; AR502)	No effect on nematode population densities	[76]
Forest trees				
*Bursaphelenchus xylophilus*	Pine trees	*Escherichia coli* (M131, M132) *Serratia marcescens* (M44)	*E. coli* and *S. marcescens* showed significant nematicidal activity (67 and 60% mortality) respectively	[77]
*M. incognita*	*Shorea* sp.; *Swietenia* sp.; *Albizia falcataria*; *Anthocephalus cadamba*; *Juglans nigra*	Bacterial isolates	Inhibited egg hatching up to 81% and mortality up to 85%	[78]
*B. xylophilus*	Pine trees	* Stenotrophomonas * and *Bacillus* sp.	Significant inhibitory activity against PWN during their developmental stages	[79]

**Table 2:** Secondary metabolites identified in endophytes and their effect on PPN.

Metabolite	Bacteria/Fungi	Nematode	References
Pregaliellalactone	*Galiella rufa*	*Meloidogyne incognita*	[98]
3-Hydroxypropionic acid	Endophytic fungi	*M. incognita*	[99]
Chlorinated oxazinane derivate (1-[(2R*,4S*,5S*)-2-chloro-4-methyl-1,3-oxazinan-5-yl] ethenone) and an epimer of the former (1-[(2R*,4S*,5R*)-2-chloro-4-methyl-1,3-oxazinan-5-yl] ethanone)	*Geotrichum* sp. (AL4)	*Bursaphelenchus xylophilus; Panagrellus redivivus*	[100]
Fusaric acid and Bikaverin	* Fusarium oxysporum * (EF119)	*B. xylophilus*	[101]
(R)-(−)-2-ethylhexan-1-ol	*Brevundimonas diminuta* (LCB-3)	*B. xylophilus*	[102]
Chaetoglobosin A	*Chaetomium globosum* (NK102)	*M. incognita*	[103]
3-methyl-1-butanol, (±)-2-methyl-1-butanol, 4-heptanone, and isoamyl acetate	*Daldinia* cf. *concentrica*	*Meloidogyne javanica*	[104]
4-hydroxybenzoic acid, indole- 3-acetic acid and gibepyrone D	*F. oxysporum* (162)	*M. incognita*	[97]
Chaetoglobosin A, chaetoglobosin B and flavipin	*C. globosum* (YSC5)	*M. javanica*	[87]

## Data Availability

Not applicable.
